# Copper and mercury induced oxidative stresses and antioxidant responses of *Spirodela polyrhiza* (L.) Schleid

**DOI:** 10.1016/j.bbrep.2020.100781

**Published:** 2020-07-17

**Authors:** Hanwant Singh, Deepak Kumar, Vineet Soni

**Affiliations:** Plant Bioenergetics and Biotechnology Laboratory, Department of Botany, Mohanlal Sukhadia University, Udaipur, Rajasthan, 313001, India

**Keywords:** Chlorophylls, Duckweed, Heavy metals, Malondialdehyde, Proline, Reactive oxygen species, CAT, catalase, Chl, chlorophyll, GPOD, Guaiacol peroxidase, HMs, heavy metal, MDA, malondialdehyde, NBT, nitro-blue tetrazolium, PUFA, polyunsaturated fatty acids, RFN, relative frond number, ROS, reactive oxygen species, SOD, superoxide peroxide, TBA, thiobarbituric acid, TCA, trichloroacetic acid

## Abstract

Duckweed is recognized as a phytoremediation aquatic plant due to the production of large biomass and a high level of tolerance in stressed conditions. A laboratory experiment was conducted to investigate antioxidant response and mechanism of copper and mercury tolerance of *S. polyrhiza* (L.) Schleid. To understand the changes in chlorophyll content, MDA, proline, and activities of ROS-scavenging enzymes (SOD, CAT, GPOD) during the accumulation of Cu^+2^ and Hg^+2^, *S. polyrhiza* were exposed to various concentrations of Cu^+2^ (0.0–40 μM) and Hg^+2^ (0.0–0.4 μM). antioxidant activity initially indicated enhancing trend with application of 10 μM Cu^+2^; 0.2 μM Hg^+2^ (SOD), of 20 μM Cu^+2^; 0.2 μM Hg^+2^ (CAT) and of 10 μM Cu^+2^;0.2 μM Hg^+2^ (GPOD) and then decreased consistently up to 40 μM Cu^+2^ and 0.4 μM Hg^+2^. In the experiment chlorophyll and frond multiplication initially showed increasing tendency and decreased gradually with the application of increased metal concentration. Application of heavy metal has constantly enhanced proline and MDA content while the maximum increase was observed with the application of 40 μM Cu; 0.4 μM Hg for proline and MDA respectively. The upregulation of antioxidant enzymes and proline reveals that *S. polyrhiza* has strong biochemical strategies to deal with the heavy metal toxicity induced by the accumulation of Cu^+2^ and Hg^+2^.

## Highlights

• Biochemical responses of copper and mercury in *Spirodela polyrhiza* *were investigated.*
• The threshold tolerance level for Cu and Hg was found < 20 μM and < 0.2 μM respectively.
• The results reveal that *S. polyrhiza* is biochemically well adapted to cope with HM toxicity induced by Cu and Hg.

## Graphical abstract

**Figure:**
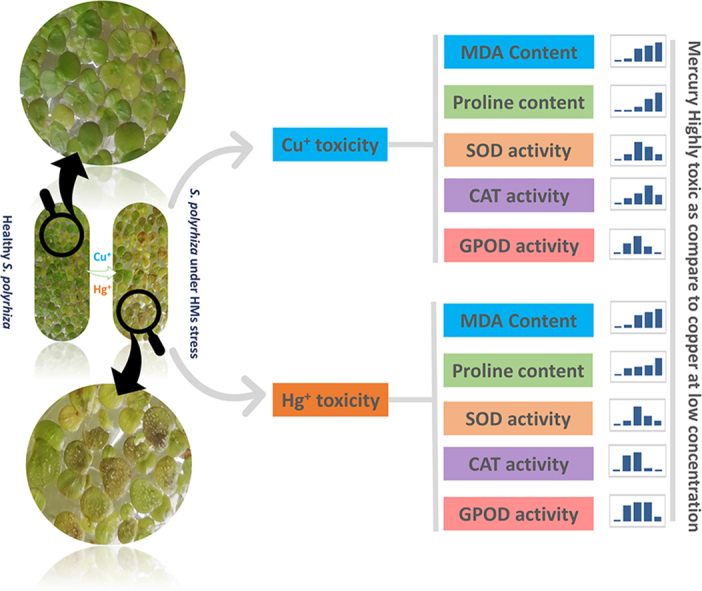

## 1 Introduction

Numerous reports have shown that Heavy metals toxicity has become one of the major environmental threats [1] due to their adverse effects on plants, animals, and soil fertility [2]. HMs include Pb, Cd, Hg, Ni, Co, Fe, Zn, Cr, Cu, Fe, As, Ag, and the Pt group elements. Several natural sources [3,4], and anthropogenic activities [[4], [5], [6], [7]]) cause significant accumulation of HMs in the ecological food chain through uptake at the primary producer level and then through consumption at consumer levels [8,9]. The heavy metal toxicity adversely affects plant tissues and can decrease plant biomass, seed germination capacity, and chlorophyll synthesis [10]. An excessive amount of heavy metal inside the plant cell can halt various cellular processes such as photosynthesis, respiration, biochemical processes, etc. [11]. In plants, heavy metal stress can trigger increased production of ROS at a particular cellular compartment such as mitochondria, chloroplast, peroxisome, etc. The increased amount of ROS into the cell can induce oxidative stress and produce lipid peroxidation, ion leakage, DNA damage, and biomolecule degradation [[12], [13], [14]]. To detoxify any deleterious reactive free radicals’ plants, naturally use most efficient mechanism via activation of antioxidant enzymes [15] such as SOD, GPOD, CAT, etc. such detoxification strategy of plant prevents cell injury and tissue damage [16,17].

Copper (Cu^+2^), is a micronutrient essential heavy metal for higher plants and algae, particularly for photosynthetic electron transport [18,19]. However, at elevated concentrations, it becomes phytotoxic, interfering in numerous biochemical and physiological processes [20,21]. The root morphology, synthesis of photosynthetic pigments, homeostasis, quantum yield, and photosynthesis are adversely modulated at high Cu^+2^ concentrations [22,23]. Mercury (Hg^+2^), a non-essential HM, is one of the severe threats to the environment and cause lethal effects on plants as it inhibits many important biological processes such as pollen germination and tube growth [24], photosynthesis [25,26], seed germination [27]and respiration. Cu^+2^ [28] and Hg^+2^ [26] both eventually enters the aquatic bodies and cause severe water contamination.

Many conventional methods such as Chemical precipitation [29], coagulation-flocculation [30], Dissolved air flotation [31], ion exchange [32], ultrafiltration [33], nanofiltration [[34], [35], [36]], reverse osmosis [37], membrane filtration, chemical precipitation, and flotation [38,39], have been used for a long time to remediate heavy metals of the water source. All these have some advantages and limitations in their use, one is more expensive [40] and secondly, they produce secondary pollutants in the environment [41,42]. During the last two decades, plant-based removal of HMs has gained worldwide recognition after the discovery of plants that can accumulate a high quantity of HMs in their tissues [[43], [44], [45]]. Several hyperaccumulator plant species have been identified, and many are still being investigated for the removal of HMs from soil and water. Among aquatic plants duckweeds [46], *Azolla* species [47], *Eichhornia crassipes* [48], *Hydrilla verticillata,* and *Pistia stratiotes* [49]are the potential candidates for phytoremediation of HMs.

Duckweeds is a group of tiny free-floating flowering plant species found in all around the world and mainly proliferate by vegetative budding found on leaf-like thallus called frond [50]. Duckweed is monocotyledon and belonging to the family Lemnaceae [51]. The family Lemnaceae involves four genera, *Lemna*, ***Spirodela***, *Woiffia*, and *Wolffiella*, among which about 37 species have been recognized so far [52]. Duckweeds have a wonderful strategy to deal with an adverse condition like low temperature by the formation of turion a dormant structure [53]. Tolerance to HMs is a crucial requirement for plant species to be used for phytoextraction [54]. Therefore, species having high potential for phytoremediation display various strategies to counter and acclimatize HMs [46,55].

Thus, by knowing the importance of Cu^+2^ and Hg^+2^, the present work designed to investigate the response to oxidative stresses, and the possibility of Cu^+2^ and Hg^+2^ phytoextraction by *S. polyrhiza* (duckweed). Furthermore, biochemical approaches were used to identify the mechanisms of HMs tolerance induced by Cu^+2^ and Hg^+^ in *S. polyrhiza* by evaluating the roles of key components such as (i) Relative frond number, (ii) chlorophyll content, (iii) MDA and free proline content (iv) oxidative stress and activities of antioxidant enzymes.

## 2 Materials and methods

### 2.1 Plant material, growth condition, and HM treatment

**Fig. 1:**
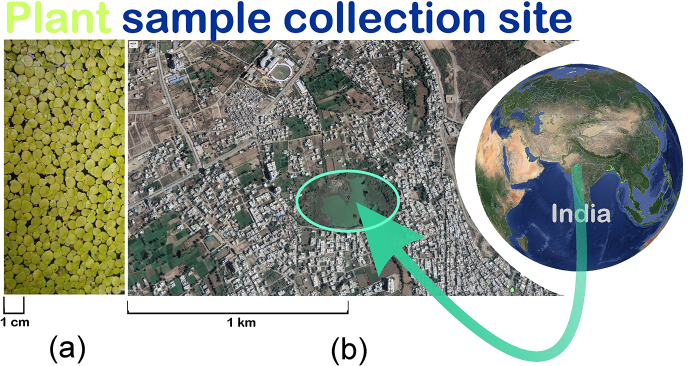
Macroscopic view of *S. polyrhiza* (a), and satellite image of plant collection site (b).

**Fig. 2:**
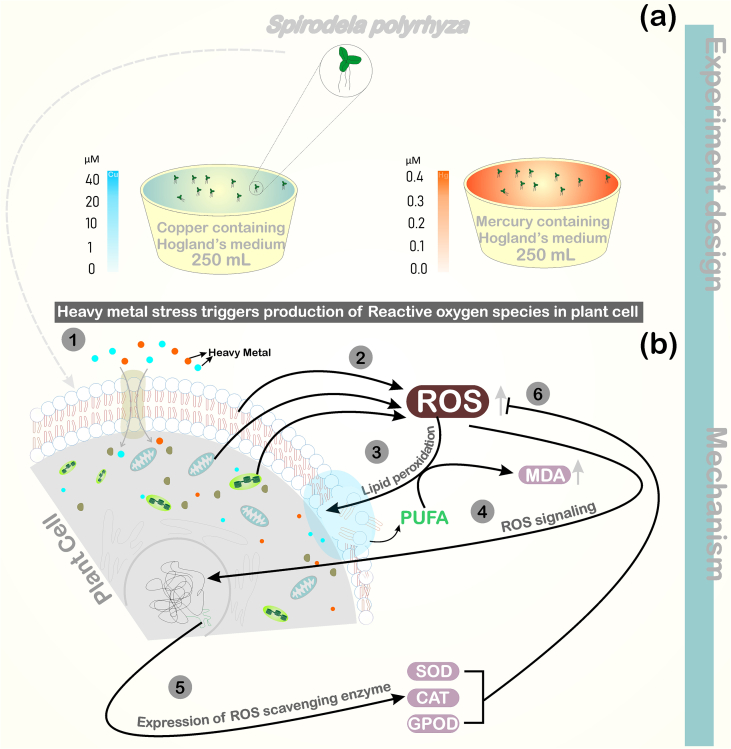
Schematic diagram of experiment design(a) and mechanism(b). HMs enters into a plant cell (1), and translocated into the various cellular compartment (Mitochondria, chloroplast, peroxisome, cell membrane, etc.) where trigger the enhanced ROS production (2), elevated amount of ROS leads to deformation of the cell membrane through lipid peroxidation and produce MDA as a byproduct of PUFA decomposition (3). Excessive ROS also acts as a signaling molecule (4) and activates the expression of ROS scavenging enzyme (SOD, CAT, GPOD, etc.) (5), which detoxifying the ROS induced cellular damage.

*S. polyrhiza* used in this investigation were collected from a freshwater pond (24°35′56.12″N, 73°42′17.79″E), in Udaipur City, India (Fig. 1). The fronds were acclimatized in 10 % Hoagland's [56] growth medium under laboratory condition as per OECD 221(6500–10000 lux light irradiance, 14-h photoperiod, and 25/20 °C day/night temperature [57].After 1 week of acclimatization of plants in medium, healthy, similar-sized fronds (about 3 g) were treated with Cu^+2^ and Hg^+2^ treatment was induced by incorporating the various concentrations of CuSO4.5 H_2_O (Sigma Aldrich, C8027, ≥98%) (0.0, 1, 10, 20 and 40.0 μM) and HgCl_2_ (HiMedia, GRM1067, 99%) (0.0,0.1, 0.2, 0.3 and 0.4 μM) in the medium (Fig. 2). The growth medium was changed every 2 d with fresh media to maintain constant heavy metal concentration and sufficient nutrients. After five-day of metal exposure, the relative frond number (RFN), concentrations of chlorophyll (Chl), MDA, and proline, activities of SOD, CAT, and GPOD were determined in the fronds of *S. polyrhiza.*

### 2.2 Determination of relative frond number

Relative frond number (RFN) was calculated by counting the fronds over 5 days on 0,1,2,3,4, and 5 (n) days [15]. RFN value was calculated as per following formula [58]-

$$RFN=\frac{frond\;number\;at\;day\;n-frond\;number\;at\;day\;0}{frond\;number\;at\;day\;0}$$

### 2.3 Determination of chl a and content

Chl *a* and Chl *b* contents were determined according to the method given by Liu et al. [59].with some amendments. 300 mg fronds were homogenized in 3.0 ml 80 % acetone. Further, the plant extract was centrifuged at 4 °C and 4000×*g* for 10 min, and the supernatant was used for chlorophyll content determination. The absorbance of Chl extract was measured spectrophotometrically (Analytikjena®Specord 200, Germany) at 645 and 663 nm.

Finally, calculations were made by using an equation and adjusted extinction coefficients given by Lichtenthaler [60].

### 2.4 Determination of MDA, proline contents

The method described by Zhao [61] was used for the determination of MDA content.300 mg fresh fronds were homogenized with pestle and mortar in 10% TCA. Homogenates were centrifuged (4000×*g* for 20 min) and the supernatant was used for MDA content determination. 2 mL aliquot of the supernatant, 2.5 mL of 0.5 % TBA in 10 %TCA was added. After centrifugation at 10,000×*g* (4 ^O^C) for 5 min, the absorbance of the supernatant was recorded at 532 and 450 nm. Lipid peroxidation was expressed as the term of MDA content in μmol g^−1^ FW.

Proline content was determined by using acid ninhydrin solution according to the method of Saradhi [62]. 300 mg fresh frond of heavy metal treated *S. polyrhiza* were homogenate in 5 ml 3% sulphosalicylic acid and after that centrifuged at 4000×*g* for 20 min. 2 ml aliquot of the supernatant was transferred in a test tube, 2 ml acetic acid, and 2 ml acid ninhydrin were mixed in a tube and boiled for 30 min. The reaction mixture containing the tube was transferred to an ice bath to stop the reaction. 4 ml toluene was added into the reaction mixture and mixed thoroughly by a vortex. The optical density of the upper toluene phase was determined at a wavelength of 520 nm.

### 2.5 Assay of antioxidant enzyme

For the preparation of enzyme extract for antioxidant enzyme assay about 300 mg heavy metal treated *S. polyrhiza* fronds (fresh weight) were homogenized in 5 ml of ice-cold potassium phosphate buffer (0.1 M, pH 7.8). The homogenate was centrifuged at 15,000×*g* (4 ^O^C) for 20 min (Remi®, India). the supernatant was separated and used as the enzyme extract.

SOD (EC 1.15.1.1) activity was assayed spectrophotometrically according to the modified method of Giannopolitis et al. [63]. The inhibition capacity of photochemical reduction of NBT by SOD was measured at 560 nm. The reaction mixture contained 100 μl, l- methionine, 100 μl NBT, 10 μl riboflavin, and 100 μl enzyme extract. Make up the volume to 3 ml by adding 0.05 M Na_2_CO_3._ The tubes containing reaction mixture were placed below white fluorescent light for 10 min after that the reaction stopped by placing the tubes in dark for 8 min and absorbance was measured at 560 nm. SOD enzyme to produce a 50 % inhibition of the reduction of NBT was expressed as one unit of SOD enzyme activity.

CAT (EC 1.11.1.6) activity was measured by the consumption of H_2_O_2_ at 240 nm according to the method of Aebi [64]. The reaction mixture containing 120 μl enzyme extract, 80 μl H_2_O_2_ (500 mM), and make final volume to 3 ml by adding 2.8 ml of 50 mM potassium phosphate buffer (pH = 7.8). The CAT activity was measured by measuring the reduction in absorbance at 240 nm as a result of H_2_O_2_ consumption.

GPOD (EC 1.11.1.7) activity was determined spectrophotometrically by measuring changes in absorbance at 436 nm for 15 s up to 5 min. Reaction mixture containing 300 μL guaiacol (1%), 1.7 ml phosphate buffer (0.05 M, pH 7.0) and 200 μL enzyme extract. the reaction started by adding 300 μL H_2_O_2._ [65]_._ The enzyme required for the transformation of the substrate in 1 min is expressed as unit enzyme activity.

### 2.6 Statistical analysis

For one-way ANOVA, SPSS® statistical package (Window Version 18.0) was used for data analysis. All statements presented in this study are at the p ≤ 0.05 levels. All data presented in the paper are the means of at least three replicates.

## 3 Results

### 3.1 Effect on relative frond number

**Fig. 3:**
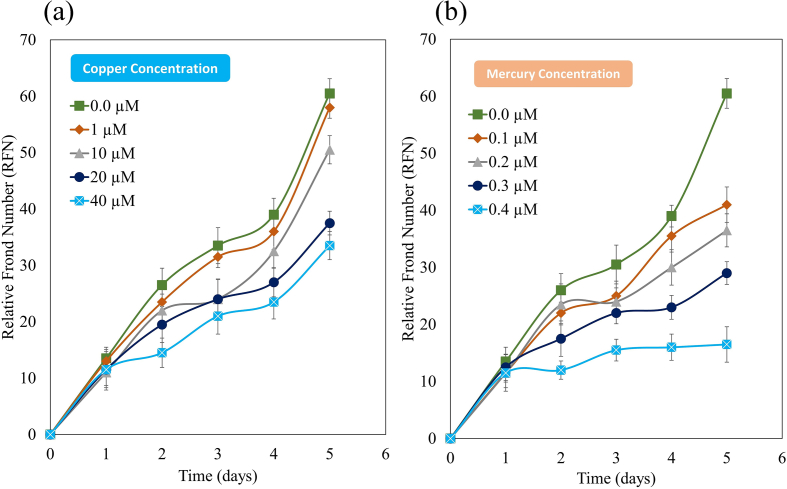
Effects of Cu^+2^ (a) and Hg^+2^ (b) on the RFN in *S. polyrhiza*.

The metals Cu^+2^ and Hg^+2^ caused irreversible damage to duckweed at concentrations of 40 and 0.4 μM respectively. The present study found that RFN decreased with an increase in Cu^+2^ and Hg^+2^ concentration in the growth medium (Fig. 3a and b). However, Cu^+2^ at low concentration (1.0 μM) could not evoke any significant changes in RFN. The RFN was significantly decreased at 40 and 0.4 μM concentrations of Cu^+2^ and Hg^+2^ respectively after 5 days of treatment.

### 3.2 Effect on chl a and chl b content

A moderate rise in Chl a and Chl b concentration (non-significant, *p* ≤ 0.05) was observed when plants were subjected to 1.0 μM Cu^+2^ (Table 1). A significant decrease in Chl a and Chl b amount was observed at 10 μM Cu^+2^. At elevated Cu^+2^ concentration (20–40 μM), the amount of Chl a and Chl b was declined gradually. Chl a reached a minimum value of 0.3513 ± 0.038 mg g^−1^ FW (66.92 % of control), while Chl b was observed to be less inhibited by Cu^+2^ and reached its minimum value 0.305 ± 0.019 mg g^−1^ FW (88.92 % of control).

**Table 1:** Mean values of the Chlorophyll *a* and chlorophyll *b* content measured in *S. polyrhiza* after exposure of five days to a medium enriched with various concentrations of copper and mercury.

Conc.(μM)	Chl *a* content (% of control)(mg g^−1^ FW)		Chl *b* content (% of control)(mg g^−1^ FW)	
Cu^+2^/Hg^+2^	Cu^+2^	Hg^+2^	Cu^+2^	Hg^+2^
Control	0.525 ± 0.022^a^(100 %)	0.531 ± 0.031^a^(100 %)	0.343 ± 0.026^a^(100 %)	0.347 ± 0.014^a^(100 %)
1/0.1	0.527 ± 0.003^a^(100.44 %)	0.506 ± 0.008^a^(95.355 %)	0.345 ± 0.002^a^(100.48 %)	0.346 ± 0.009^a^(99.61 %)
10/0.2	0.452 ± 0.018^b^(86.03 %)	0.495 ± 0.018^ab^(93.158 %)	0.339 ± 0.010^ab^(90.57 %)	0.328 ± 0.032^ab^(94.34%)
20/0.3	0.397 ± 0.041^b^ (75.619 %)	0.457 ± 0.033^b^ (86.127 %)	0.311 ± 0.009^bc^ (90.58 %)	0.317 ± 0.006^ab^(91.37 %)
40/0.4	0.351 ± 0.038^c^(66.921%)	0.334 ± 0.026^c^(59.824 %)	0.305 ± 0.019^c^(88.92 %)	0.290 ± 0.036^b^(83.40%)

$\bar{x}$ ± S for three replicate measurements at a 95% level of confidence. Different letters indicate a significant difference (*P* ≤ 0.05).

**Fig. 4:**
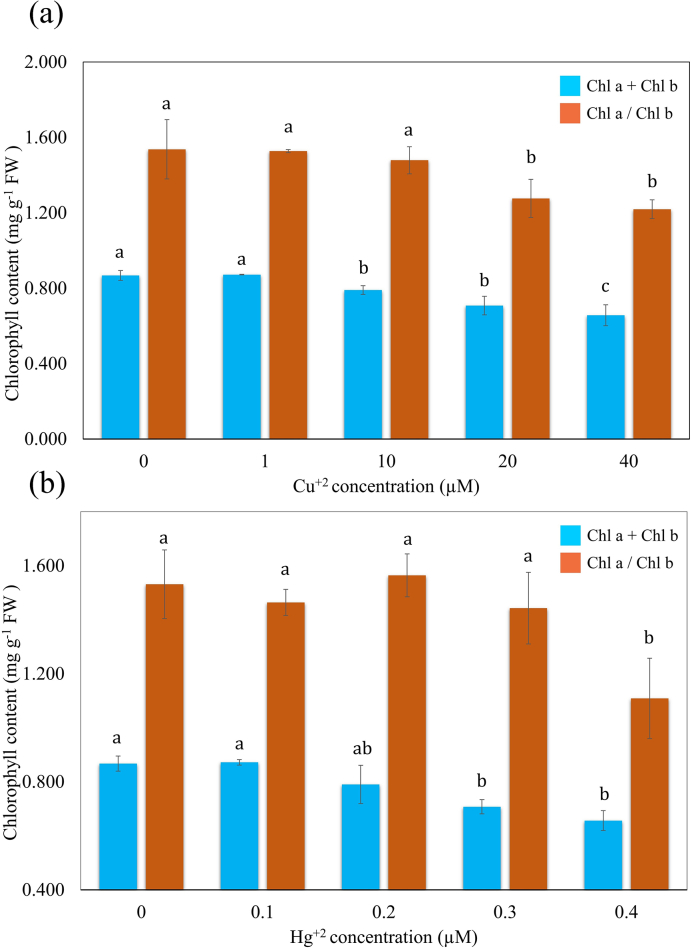
Effects of Cu^+2^ and Hg^+2^ on the total Chl (a + b) and Chl a/b ratio. Values are presented in the average of three replicates and standard errors are represented by error bars. Different characters indicate significant differences among the results (*p* ≤ 0.05).

Hg^+2^ affected the concentration of Chl a and Chl b in *S. polyrhiza* (Table 1). A concentration-dependent reduction in Chl a was noted, when plants were exposed to 0–0.4 μM Hg^+2^. Low Hg^+2^ concentration (0.1–0.2 μM) could not evoke any significant decline in Chl a concentration. Thereafter, a marked reduction of control in Chl a content was observed at increased Hg^+2^ concentration. Hg^+2^ at 0.4 μM, reduced Chl a 59.824% of control. In contrary to Chl a, Chl b was found more stable under Hg^+2^-induced HM stress. Total Chl a + Chl b declined in a concentration-dependent pattern of Cu^+2^ and Hg^+2^. At a higher concentration of Cu^+2^ (40 μM), the value of total Chl was decreased significantly and reached to 75.610 % of control (0.656 ± 0.055 mg g^−1^ FW) (Fig. 4a). In the present study, Hg^+2^ led a pronounced effect on the total Chl content of *S. polyrhiza*. Hg^+2^ at 0.4 μM, declined the total Chl content 69.172 % of control (0.607 ± 0.036 mg g^−1^ FW) in the fronds of *S. polyrhiza* (Fig. 4b).

The Chl a/Chl b, which reflects the effectiveness of light-harvesting complexes, drastically altered in both Cu^+2^ and Hg^+2^ treatment. At 40 μM of Cu^+2^, the value of the Chl a /Chl b was 1.22 ± 0.050 (79.33 % of control) observed (Fig. 4a), however, a trace amount of Hg^+2^ lowered the Chl a/Chl b (72.465 % of control) in the fronds of *S. polyrhiza* (Fig. 4b). The present study indicates that Hg^+2^ is more toxic even at lower concentrations (0.0–0.4 μM) as compared to increased Cu^+2^ concentration (0.0–40 μM).

### 3.3 Effect on MDA, proline contents and ROS-scavenging enzymes

**Fig. 5:**
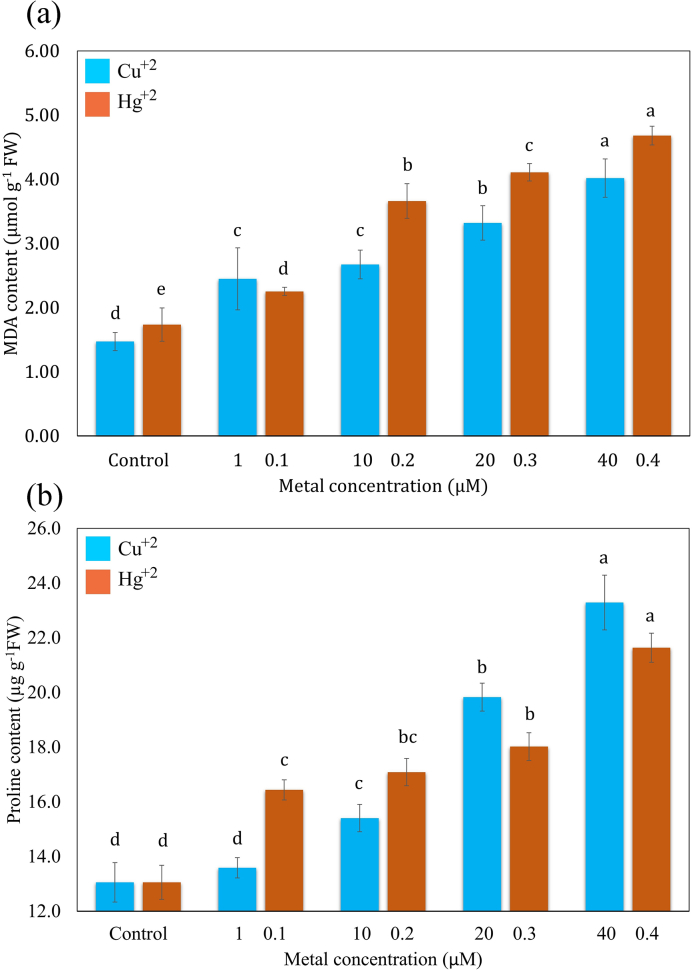
Effects of Cu^+2^ and Hg^+2^ on the MDA (a) and free proline content(b). Values are presented in the average of three replicates and standard errors are represented by error bars. Different characters indicate significant differences among the results (*p* ≤ 0.05).

MDA, a product of lipid peroxidation, has been used as a marker of cellular damage caused by environmental stresses. In the present study. HM-stress induced by different concentrations of Cu^+2^ and Hg^+2^ stimulated the accumulation of MDA in the frond of *S. polyrhiza* (Fig. 5a). MDA exhibited a maximum value of 4.018 ± 0.30 μmol g-1 FW and 4.681 ± 0.150 μmol g-1 FW at highest concentrations of Cu^+2^ (40 μM) and Hg^+2^ (0.4 μM) respectively. Likewise, proline content enhanced by increasing the concentration of Cu^+2^ and Hg^+2^ in the medium. At highest concentrations of Cu^+2^ and Hg^+2^, the proline content increased remarkably and reached to 23.325 ± 1.0 μg g^−1^ FW and 21.634 ± 0.530 μg g^−1^ FW respectively.

**Fig. 6:**
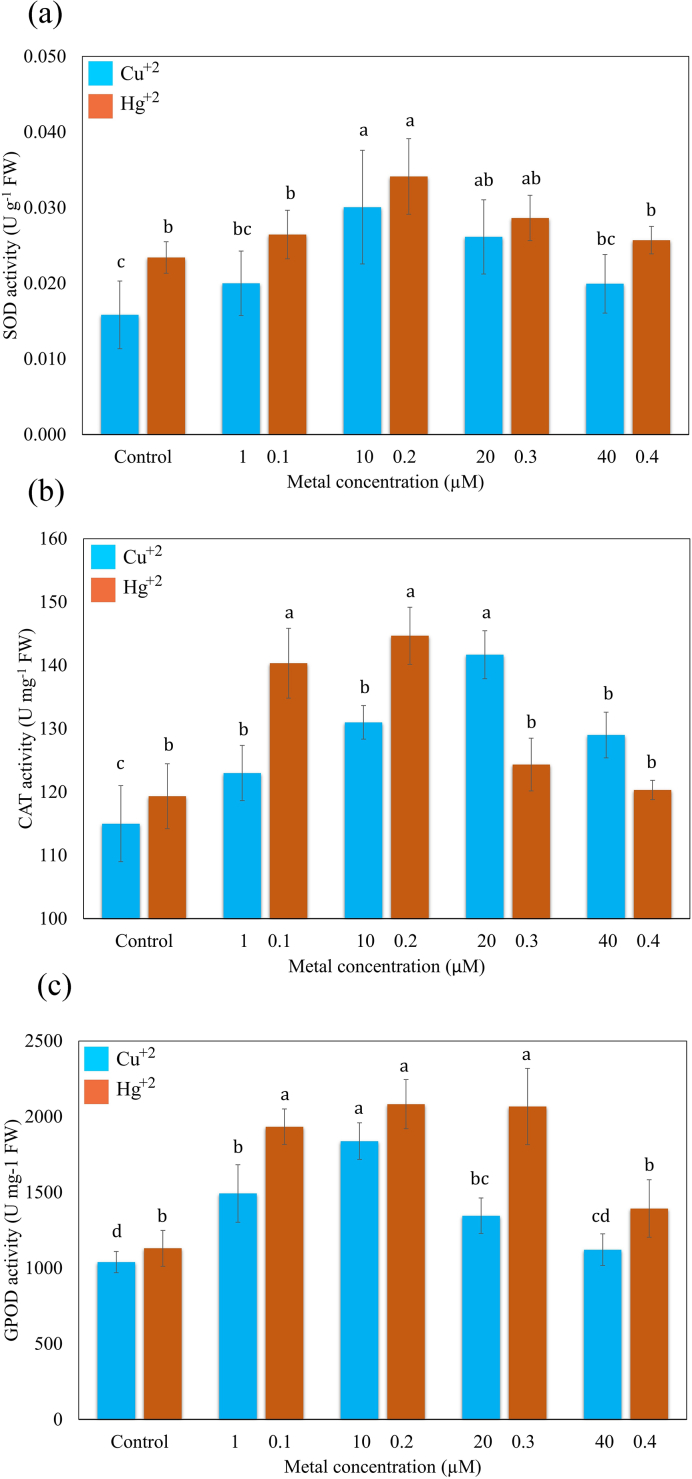
Effects of Cu^+2^ and Hg^+2^ on the SOD (a), CAT (b), and GPOD activity (c). Values are presented in the average of three replicates and standard errors are represented by error bars. Different characters indicate significant differences among the results (*p* ≤ 0.05).

In response to HM-stress activated by Cu^+2^ and Hg^+2^, the activities of ROS-scavenging enzymes significantly modulated (Fig. 6 a,b,c). The SOD activity increased at lower Cu^+2^ (1.0 μM) concentration until they reached a peak, before decreasing at higher concentration. An increase in enzyme activity (187.91 % of control) was recorded in the fronds subjected to 10 μM Cu^+2^. Similarly, SOD activity upregulated 145.86% of control at 0.2 μM Hg^+2^ and then declined progressively with increasing Hg^+2^ concentration up to 0.4 μM. However, the SOD activity remained higher over control at the maximum concentrations of Cu^+2^ (40 μM) and Hg^+2^ (0.4 μM) (Fig. 6a).

CAT activity increased significantly with the progression of Cu^+2^ stress up to 20 μM. At a higher concentration of Cu^+2^, the enzyme activity was remarkably downregulated, but it remained high when matched to control. Similarly, in fronds treated up to 0.2 μM Hg^+2^, the CAT activity increased linearly (121.56 % of control) and thereafter reached the almost control value with the progression of Hg^+2^ concentration into the medium (Fig. 6b).

*S. polyrhiza* displayed a wide variation in GOPD activity when treated to different concentrations of Cu^+2^ and Hg^+2^. A remarkable increase in enzyme activity was observed in plants growing in medium containing 10 μM Cu^+2^ (176.93% of control). The high concentration of Cu^+2^ caused an inhibitory effect on enzyme activity. On the contrary, GOPD activity markedly upregulated at 0.1 μM (171.09% of control), and then gradually increased up to 0.3 μM Hg^+2^. Elevated Hg^+2^ severely inhibited the GOPD activity in the fronds of *S. polyrhiza* (Fig. 6c).

## 4 Discussion

### 4.1 Effect on relative frond number

The present study found that Growth parameters, in terms of relative frond number decreases with an increase in growth medium's metal concentration, were affected more strongly by Hg^+2^ than by Cu^+2^. A similar effect has been reported by Xue at al [66]. A decline in RFN is considered as an indicator of environmental stress [67,68].

### 4.2 Effect on chl a and chl b content

In the present study, exposure to Cu^+2^ and Hg^+2^ severely affected Chl a, Chl b content, total Chl a + Chl b, and Chl a/Chl b ratio of *S. polyrhiza*. Chl is highly sensitive to the oxidative stress induced by environmental changes and heavy metals [69,70].

According to Manios et al. and Ewais et al. [69,71], changes in the chlorophyll content are important parameters that should always be taken under consideration when studying the plant responses to environmental stresses. In the present study, as compared to Cu^+2^, Hg^+2^ provoked more toxic effects on Chl amount in *S. polyrhiza*. HM-induced diminution in total Chl content has been attributed to the activation of Chl degradative enzymes and inhibition of enzymes involved in Chl biosynthesis [72]. The replacement of Mg^+2^ in Chl by Cu ^+2^and lipid peroxidation of chloroplast membrane could also be a major cause for pigment loss in *S. polyrhiza* [73,74]. Destruction in chlorophyll ultrastructure altered the shape and size of thylakoid which is responsible for Chl a and b content in plants [75]. Chl was found more sensitive to Hg^+2^ induced HM stress. Loss in Chl occurred due to the rapid degradation of Chl a. The Chl a/Chl b ratio, which is used as a stress indicator, slightly decreased with increment of Cu^+2^ stress [15]. On the other hand, Hg^+2^ severely affects the Chl ratio in the frond of *S. polyrhiza*. On the contrary, increased Chl a/Chl b ratio, an indicator of change in the PSII/PSI ratio, has been reported in stressed leaves of spinach [76].

### 4.3 Effect of MDA, proline content and ROS-scavenging enzymes

MDA a byproduct of PUFA decomposition and its elevated level indicates plants are under an increased level of antioxidant stress [77]. MDA (a cytotoxic product of lipid peroxidation) level is believed to be the best measure of lipid peroxidation status and cell membrane damage induced by ROS production [78,79]. In the present study, an increased level of MDA suggests that Cu^+2^ and Hg^+2^ stimulated MDA production through the excessive generation of ROS, resulting in increased lipid peroxidative products and oxidative stress in the fronds of *S. polyrhiza.* A similar finding was obtained in the *Ceratophyllum demersum* L [80].

Accumulation of free proline is considered as a tolerance strategy of plants in response to HM exposure [81,82]. Physiologically, proline facilitates plant to maintain water potential and homeostasis [83] and protect them from adverse effects of ROS [84]. The accumulation of proline might be associated with the reduction of negative impacts of Cu^+2^ and Hg^+2^ on plant growth and indicates one of the biochemical strategies to cope with HM stress in *S. polyrhiza.*

ROS- scavenging enzymes are sensitive biochemical parameters to probe the plant responses to environmental stresses. Upregulation of ROS- scavenging enzymes is one of the protective mechanisms to allow plants to overcome metal toxicity [82]. The activated antioxidant enzyme defense system can reduce the harmful ROS by modulating the K^+^ efflux and electron transport chain [85,86]. SOD is a key component of the antioxidant system in plants that dismutase two superoxide radicals (O_2_^-^) to O_2_ and H_2_O_2_. CAT and GPOD are important ROS scavenging enzymes that convert H_2_O_2_ into water and oxygen and regulates H_2_O_2_ levels in the cellular compartment [87]. Like MDA and proline, the activities of SOD, CAT, and GPOD detected in the fronds improved consistently under increasing Cu^+2^ and Hg^+2^ toxicity. Increased activities of antioxidant enzymes indicate that *S. polyrhiza* is biochemically well adapted to detoxify ROS accumulated during the exposure of various concentrations of Cu^+2^ and Hg^+2^. It was reported by many current researchers that antioxidant enzyme activities enhanced under lower heavy metal concentration, and then decreased at higher metal stress [[88], [89], [90]] HM-stimulated activities of antioxidant enzymes have already been reported by many researchers in other aquatic plants used for phytoremediation [61,77,82,91]. In plants, both enzymatic and non-enzymatic defense system were performed to overcome heavy metal stress [92]. The study proves with evident results that the antioxidant enzyme system was activated in plants under Cu and Hg at 1–20 μM and 0.1–0.2 μM.

## 5 Conclusion

In this paper, ecotoxicological effects and biochemical responses to Cu^+2^ and Hg^+2^ stress in aquatic angiosperm *S. polyrhiza* are discussed. Results conclude that plant growth characteristic (RFN), chlorophyll content, and antioxidant enzyme activity (SOD, CAT, and CAT) were enhanced at the lower concentration of heavy metal to control plants. Chl pigments, MDA (malondialdehyde), proline, and activities of ROS-scavenging enzymes (SOD, CAT, GPOD) are more sensitive to Hg^+2^ toxicity as compared to Cu^+2^. The threshold level for Cu^+2^ and Hg^+2^ was found < 20 μM and < 0.2 μM respectively in *S. polyrhiza*. The current investigation has provided important evidence that illuminates the mechanism of Cu^+2^ and Hg ^+2^ toxicity in duckweed *S. polyrhiza*. Although there is more research required in the field of phytoremediation to explore and this study revealed that the plant *S. polyrhiza* is a decent metal tolerant plant and can be used for phytoremediation.

## Funding

This research did not receive any specific grant funding from agencies in the public, commercial, or not-for-profit sectors.

## Authors contribution

H.S. conceived the idea and designed the experiment. H.S. and D.K. performed the experimental work and data analysis. V.S. supervised the experiment work. H.S. wrote the initial manuscript and prepared figures. H.S., D.K., and V.S. contributed to discussing, reviewing, and approving the final version of the manuscript for publication.

## Declaration of competing interest

The authors declare that they have no known competing financial interests or personal relationships that could have appeared to influence the work reported in this paper.
